# Intralesional Injection of Bone Marrow Aspirate Concentrate for the Treatment of Osteonecrosis of the Knee Secondary to Systemic Lupus Erythematosus: A Case Report

**DOI:** 10.3389/fbioe.2020.00202

**Published:** 2020-03-20

**Authors:** Dimitrios Kouroupis, Amir F. Ahari, Diego Correa, Riam Shammaa

**Affiliations:** ^1^Department of Orthopaedics, UHealth Sports Medicine Institute, University of Miami Miller School of Medicine, Miami, FL, United States; ^2^Canadian Centers for Regenerative Therapy, Toronto, ON, Canada; ^3^Diabetes Research Institute & Cell Transplant Center, University of Miami Miller School of Medicine, Miami, FL, United States; ^4^Department of Family and Community Medicine, University of Toronto, Toronto, ON, Canada

**Keywords:** osteonecrosis, systemic lupus erythematosus, bone marrow aspirate concentrate, mesenchymal stem cells, endothelial progenitor cells, cellular therapy, bone regeneration

## Abstract

**Case:** An 18-year-old female patient with Systemic Lupus Erythematosus (SLE) and corticosteroid-associated extensive bilateral symptomatic knee Osteonecrosis (ON) (Ficat IV), treated with sequential intralesional injections of autologous bone marrow aspirate concentrate (BMAC) under ultrasound guidance. At 3 months, pain was almost absent (VAS) and KOOS/WOMAC showed significant improvement sustained up to 24 months. At 12 months MRI indicated bone maturation, significantly reduced BM edema and subchondral fluid volume, and no collapse/fragmentation signs.

**Discussion:** The clinical and imaging significant improvement observed in this patient suggests that BMAC intralesional injections effectively restored the compromised bone structure. After larger studies, this technique can become an alternative to decompressing surgery for ON cases.

## Introduction

Osteonecrosis (ON) or avascular necrosis defines a compromised blood supply of a bone area, which leads to localized necrosis and tissue death. Secondary ON is prevalent in younger populations and it associates with various medical conditions including systemic lupus erythematosus (SLE) (Kopecky et al., 1991; Aranow et al., 1997). SLE patients with early corticosteroid side-effects show the highest risk for ON development (Motomura et al., 2005; Nevskaya et al., 2017).

Non-operative management to operative procedures have been suggested for ON with variable clinical outcomes (Hungerford and Jones, 2004). Current treatment for the symptomatic, advanced disease is mainly surgical and generally involves a total joint replacement (Salvati and Cornell, 1988), as non-surgical therapies typically generate suboptimal outcomes (satisfactory in only ~19% of patients) (Mont et al., 1996). In an effort to prevent the progression of ON and potentially reverse the vascular compromise and secondary bone destruction, studies have investigated the use of Mesenchymal Stem Cells (MSC)-based therapy as a viable approach (reviewed in Perez et al., 2018) (Li et al., 2014). Bone marrow aspirate concentrate (BMAC) is a heterogeneous mix of multiple cellular phenotypes, including bone marrow-derived MSC (Holton et al., 2016; Imam et al., 2017) and BMAC implantation into the necrotic zone has been shown to promote bone repair by reconstructing a micro-environment of osteoblast differentiation and vascular proliferation *in vivo* (Hernigou and Beaujean, 2002; Zhao et al., 2012; Calori et al., 2014; Hernigou et al., 2015, 2016).

In the present case report, we applied sequential autologous BMAC intralesional injections to treat a young female patient with SLE and corticosteroid-associated bilateral knee ON, following up her clinically up to 2 years.

## Statement of Informed Consent

In our institution, Institutional Review Board waiver is provided for case reports. Signed informed consent for participation in the study was obtained.

## Case Report

### History

We present the case of a 18-year-old female with 10 years history of SLE referred to pain clinic for progressive, localized, dull and non-irradiated pain in both knees for over 12 months, with difficulty weight bearing particularly on the right side leading to limping and impaired walk. The pain was constant during the day and often at night, aggravated during walk and stair climbing, and partially relieved with rest. No neurological symptoms including numbness or paresthesias in lower extremities or bowel and bladder dysfunction were reported. One year before BMAC treatment, the patient was neither a drinker nor smoker, and did not report any noticeable weight change.

The patient was diagnosed in 2009 with SLE and when first seen was on a protocol of 10 mg of prednisone daily. SLE flare ups endured for periods of 4 weeks, then reduced gradually over 4 weeks and then halted. Patient had an average of 1 flare up every 6 months for a total average annual cumulative dose of 784 mg/years. Past medical history included glucocorticoid induced osteoporosis, cataracts and macrophage activation syndrome. There was no known history of significant traumas to the knees. Patient's past surgical history was negative. Patient's medications were hydroxychloroquine 200 mg po daily, levetiracetam 750 mg po twice a week for SLE related seizures, prednisone 10 mg po once daily, calcium carbonate 1,250 mg po twice daily, cholecalciferol 2000 IU po daily, ferrous fumarate 300 mg po twice daily, ramipril 7.5 mg po as needed when elevated blood pressure. Before the treatment patient had Erythrocyte Sedimentation Rate: 34 mm.h, C-Reactive Protein: 3.5 mg/L, Complement component 3: 1.2 g/L, Complement component 4: 0.23 g/L, ANA titers within normal limits, dsDNA: 13.4 IU/ml, Rheumatoid factor autoantibody: 10 μ/ml, and anti-Cyclic Citrullinated Peptide: 8 μ/ml.

### Physical Examination

Physical examination of the lower extremities showed swelling in the right knee with bilateral pronounced effusion in both knees. Palpation revealed extreme generalized tenderness in the right knee more pronounced than the left. Mobility of the right knee showed a reduced range of motion (ROM) with a lack of 70 degrees on flexion and 25 degrees on extension, inducing pain with further movement. The left knee showed neither active nor passive limitations. Muscle weakness was noted bilaterally, however, power was 5/5 bilaterally on flexion and extension. No sensory or reflex alterations were found. No joint stability (ligament) issues were observed in either knee. Upper and lower joints were in normal limits.

### Diagnostic Studies and Treatment

Based on the underlying SLE, the sustained corticosteroid therapy and the clinical presentation, the patient was suspected to have developed secondary ON in both knees. The diagnosis was confirmed by MRI, which showed extensive subchondral fluid on both lateral femoral condyles and anterior weight-bearing surfaces of the medial femoral condyles, more pronounced in the right knee. These findings were associated with impending collapse and fragmentation of the articular surface. Therefore, extensive ON involving bilateral medial and lateral femoral condyles was confirmed and staged as Ficat IV. Furthermore, areas of bone infarction in the distal femoral diaphysis and proximal tibial diaphysis were also observed, with no evidence of ON concerning the subarticular proximal tibia. Based on an initial clinical assessment, the right knee was confirmed as more advanced ON than the left (Supplemental Video 1).

**Figure 1:**
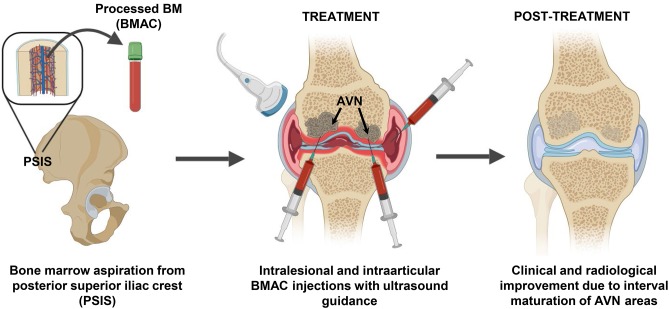
BMAC intervention strategy to treat femoral head ON. Bone marrow (BM) aspiration was performed from the posterior superior iliac crest area and the BM aspirate was concentrated (BMAC) using a commercial filtration system to concentrate the mononuclear fraction containing BMSC and BM-EPC. Five sequential intralesional and intraarticular BMAC injection sessions were performed in both knees using ultrasound guidance. Twelve months post-treatment radiological improvements were observed in previously ON areas that supported the positive clinical progression.

Bone marrow (BM) was aspirated five times (initial three times for the right knee injection and two final for the left knee injections), with intervals of 1–2 weeks and alternating the harvesting site. Under sterile conditions, the BM aspiration site (Posterior Superior Iliac Spine—PSIS) was marked upon visualization with ultrasound guidance, followed by injection of 2% lidocaine in the soft tissue and periosteum (Figure 1). The BM tissue was collected using a 14G trocar needle, with an entry point created with the introducer needle and the bone drilled through the periosteum and cortex into the spongy bone into a depth of 4 cm. Subsequently, BM was aspirated slowly at a rate of 1–2 cc per level and withdrawn gradually using heparinized syringes. The aspirated BM (i.e., BMA) was then passed through a customized size-based cell filtration system (Chondrostem device, manufactured by CCRT, Toronto, Canada. Patent pending) similar to other concentrators (Ito et al., 2009; Otsuru et al., 2013) to enrich the mononuclear cells (MNCs) fraction, especially the mesenchymal stem/stromal cells (MSC) component present within that fraction and described as the culture-expanded progeny of *in vivo* connective tissue progenitors (CTP) (Sivasubramaniyan et al., 2018).

The injections were performed under local anesthesia, sterile conditions and ultrasound guidance, using a 18-gauge 3.5-inch needle inserted 2 cm deep into the condyle bone (Figure 1). The processed BMAC was injected in five sessions for right and left knees independent treatments (Table 1), including two intra-articular injections only to the right knee given its early signs of articular cartilage involvement.

**Table 1:** Summary of BMAC therapeutic sessions with locations and volumes applied.

**Session**	**Treated knee**	**Sites injected**	**Volume aspirated before processing**	**Volume injected**
1	Right	Medial condyle	40 cc	21 cc
		Intra-articular space		5 cc
2	Right	Lateral condyle	60 cc	25 cc
		Intra-articular space		5 cc
3	Right	Medial condyle	40 cc	10 cc
		Lateral condyle		10 cc
4	Left	Medial condyle	40 cc	9 cc
		Lateral condyle		8 cc
5	Left	Medial condyle	40 cc	11 cc
		Lateral condyle		11 cc

**Figure 2:**
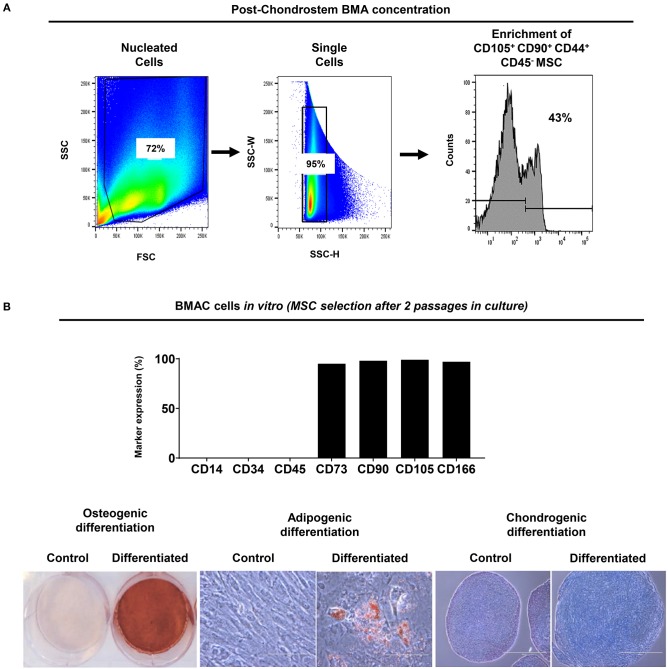
BMA enrichment using filtration system. **(A)** CD45^−^ CD44^+^ CD90^+^ CD105^+^ MSC/EPC subpopulation was enriched after BMA concentration. Isotype controls were used to exclude background staining. **(B)** Upon BMAC seeding *in vitro*, passage 2 cultures showed MSC-related phenotypic profile and tripotential differentiation capacity. Generated MSC cultures were negative for CD14, CD34, CD45, and highly positive (>90%) for CD73, CD90, CD105, CD166 markers. Differentiation was evident on day 21 for osteogenesis by mineral deposit (Alizarin Red staining), for adipogenesis by lipid accumulation (Oil Red staining), and for chondrogenesis by glycosaminoglycan production (Alcian Blue staining). Control cultures showed no differentiation capacity for all inductions tested.

*in vitro* data showed that Chondrostem filtration result in CD45^−^CD44^+^CD90^+^CD105^+^ MSC/EPC subpopulation enrichment (43% of total) (Figure 2A). MSC-related phenotypic profile and tri-potentiality (bone-fat-cartilage) was established in BMAC cells cultured for two passages *in vitro* (Figure 2B).

The patient was followed-up 1 week after each procedure at the same time with the following injection, then at 1, 3, 6, 9, and 12 months while undergoing post-surgical rehabilitation. At each visit joint effusions, swelling and tenderness to palpation at the joint line level, and range of motion (ROM) were assessed. Patient-reported outcomes measurement (PROM) were assessed using clinically validated scoring systems (VAS, KOOS, and WOMAC).

**Figure 3:**
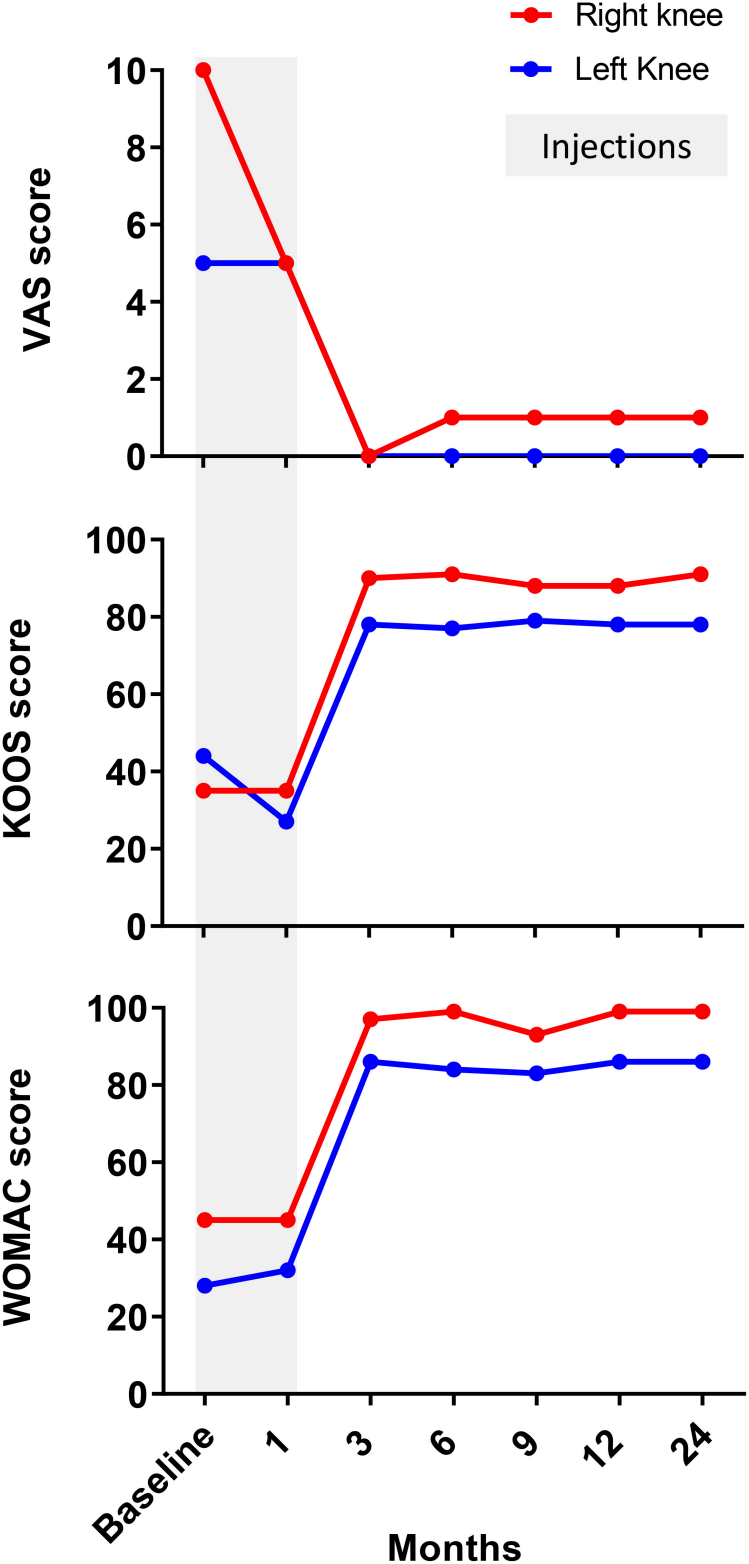
Evaluation of patient-reported outcomes measurement (PROM). Patient had significantly improved VAS, WOMAC and KOOS between 1 and 3 months after BMAC injections for both knees. Thereafter, all clinically validated scores showed stable values between 3 and 12 months supporting the positive clinical progression.

**Figure 4:**
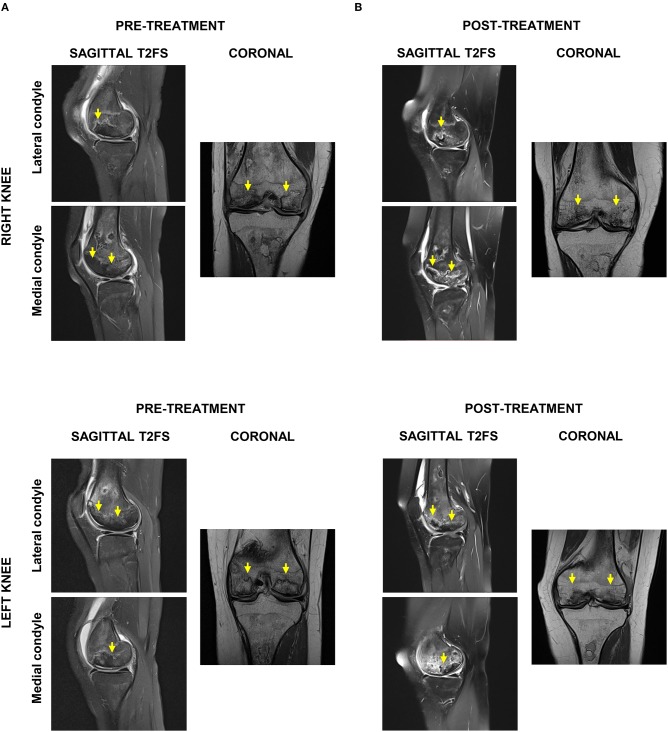
Radiological evaluation of ON affected areas by magnetic resonance imaging (MRI). **(A)** Pre-treatment sagittal and axial T2FS MRI indicated extensive bilateral ON and bone infarction, particularly of lateral femoral condyles and anterior weight-bearing surfaces of the medial femoral condyle. **(B)** Post-treatment sagittal and axial T2FS MRI showed in both knees bone maturation in ON areas, less BM edema, and reduced subchondral fluid.

Four weeks after the final procedure pain was almost absent on both sides (1–2/10), with weight-bearing the left leg (not the right one) and able to walk with crutches. On examination, there was only mild effusion on the left knee with a full ROM, whereas the right side had limited 10 degrees on extension and 35 degrees on flexion. At 6 months, the patient was pain-free (0/10 on the left and 1/10 on the right side) with a full ROM on both sides. She was able to bear weight, walk and even dance without the support of crutches. Her improvement continued until 9 and 12 months, when she was pain-free with full ROM and with the ability to run (Supplemental Video 2). Clinically, the PROM showed significant improvement over time, starting right after the end of the sequential therapy one (1) month after its start and for a 24-months follow-up (Figure 3). MRI at 12 months showed bone maturation (with granulation tissue) in active ON areas, less marked BM edema bilaterally and significant reduction in subchondral fluid, which proved radiological improvement supporting the positive clinical progression. With exception of the lateral condyle of the right knee, which showed persistent irregularities and fragmentation/collapse of the central and posterior weight bearing surfaces, the rest of structures did not show signs of collapse or fragmentation (Figure 4). At 24 months follow-up the patient was stable in terms of pain and function, able to cycle and dance, no effusion in the knees with full active and passive range of motion on physical examination.

## Discussion

Present patient developed symptomatic ON of both knees after prolonged corticosteroid administration to control SLE progression. The diagnosis was confirmed by appearance of sclerosis and/or subchondral fracture on MRI images, particularly of both lateral femoral condyles and anterior weight-bearing surfaces of the medial femoral condyle. Moreover, fluid deep to subarticular bone was observed which is consistent with impending collapse and fragmentation.

The application of cell-based therapy to treat ON was originally suggested by Hernigou and Beaujean (2002) with the premise that no other treatments could restore the structure of necrotic bone (Holton et al., 2016; Imam et al., 2017). Studies have tested the BMAC safety and efficacy for bone defect treatment, demonstrating superiority to bone grafts with fewer complications (Fayaz et al., 2011; Jäger et al., 2011; Imam et al., 2017). In the present case study, intra-lesional BMAC was serially injected in both knees. However, understanding the secondary involvement of gradual articular cartilage deterioration to osteonecrosis progression and evaluating the signs of articular cartilage deterioration in the right knee, two intra-articular BMAC injections were performed only in the right knee to prevent any further articular surface damage. Twelve months post-treatment, clinical improvement was paralleled with MRI evidence of bone maturation in previously necrotic areas and no further signs of collapse or fragmentation in both knees. The clinical improvement was maintained further to 24 months, with no changes in the clinical scores. We ascribe the therapeutic success of intralesional BMAC injections as a source of connective tissue progenitors (CTP) and endothelial progenitor cells (EPC) populations, which together may restore the compromised vascular network at the necrotic bone sites. CTP reside in the postnatal BM cavities (Mendez-Ferrer et al., 2010; Tormin et al., 2011; Levesque, 2013; Mendelson and Frenette, 2014), can differentiate into bone after *ex vivo* manipulation of their culture-expanded progeny (i.e., Mesenchymal Stem/Stromal Cells—MSC) (Sivasubramaniyan et al., 2018), while exhibiting strong angiogenic properties (Caplan Arnold and Correa, 2011; Churchman et al., 2013; Bianco and Robey, 2015; Castro-Manrreza and Montesinos, 2015; Jones et al., 2016; Kouroupis et al., 2018). Although, CTP represent a small fraction of total mononuclear cells (MNC) isolated from BM tissue ranging 0.01-0.1% (Jones and McGonagle, 2008) BMAC processing can result in up to 13.000-fold enrichment (Dragoo and DeBaun, 2017; Schäfer et al., 2019). On the other hand, BM constitute a rich source of EPC (Hristov et al., 2003). EPCs can initiate neovascularization (Asahara et al., 1997, 1999; Schatteman and Awad, 2004) and their intraoperative isolation at high yields from BMAC is feasible (Kolvenbach et al., 2010; Essl et al., 2013). In addition, BMAC promotes *in vivo* bone regeneration by bringing platelets and growth factors such as PDGF-BB and VEGF to the injury site (Hernigou et al., 2005; Holmes et al., 2007; Zhong et al., 2012; Gary et al., 2013). Therefore, BMAC application (containing both BM-MSC and EPC populations) becomes advantageous to promote simultaneously neovascularization and bone regeneration in ON lesions.

## Conclusion

Intralesional, ultrasound-guided, direct injections of autologous BMAC effectively treated areas of bone with secondary ON, inducing reversal of necrotic areas and inducing clinical improvement in this patient, thus potentially becoming an alternative to decompressing surgery after larger studies.

## Data Availability Statement

All datasets generated for this study are included in the article/Supplementary Material.

## Ethics Statement

Ethical review and approval was not required for the study on human participants in accordance with the local legislation and institutional requirements. The patients/participants provided their written informed consent to participate in this study.

## Author's Note

This study was performed at the Canadian Centers for Regenerative Therapy in Toronto, Canada.

## Author Contributions

All authors listed have made equal substantial, direct and intellectual contribution to the work, and approved it for publication.

### Conflict of Interest

The authors declare that the research was conducted in the absence of any commercial or financial relationships that could be construed as a potential conflict of interest.

## Supplementary Material

Supplemental Video 1 Patient walking in crutches pre-treatment, showing limitations exacerbated in the right leg.

Supplemental Video 2 Patient walking 12 months post-treatment, evidencing significant improvement (no limitations).
